# A Holistic Review on the Current and Future Status of Biology-Driven and Broad-Spectrum Therapeutic Options for Medulloblastoma

**DOI:** 10.7759/cureus.23447

**Published:** 2022-03-24

**Authors:** Ariana Pritha, Richard Anderson, David E Anderson, Theodore Nicolaides

**Affiliations:** 1 Research, Health Sciences Center (HSC) Neurosciences, University of New Mexico School of Medicine, Albuquerque, USA; 2 Pediatric Neurosurgery, Goryeb Children's Hospital, Morristown Medical Center, Morristown, USA; 3 Immunology, Variation Biotechnologies Inc. (VBI) Vaccines, Boston, USA; 4 Pediatric Hematology-Oncology, Pediatric Neuro-Oncology, Stephen D. Hassenfeld Children's Center for Cancer and Blood Disorders, New York City, USA

**Keywords:** autologous stem cell therapy, pediatric populations, cns tumor, immunotherapy, medulloblastoma

## Abstract

With a thorough investigation of the etiology of medulloblastomas, a comprehensive review was done to categorize available clinical trials in order to discuss the future potential of breakthroughs in treatment options. The pertinent issues of medulloblastoma therapy with radiation being inapplicable to children under the age of 3, and therapies causing toxicity are detailed and discussed in the context of understanding how the current therapies may address these suboptimal treatment modalities. This study aggregated published studies from the US government clinical trials website and filtered them based on their direct treatment towards medulloblastomas. Thirty-two clinical trials were applicable to be analyzed and the treatment mechanisms were discussed along with the efficacy; molecular groupings of medulloblastomas were also investigated. The investigated therapies tend to target sonic hedgehog (SHH)-subtype medulloblastomas, but there is a necessity for group 3 subtype and group 4 subtype to be targeted as well. Due to the heterogeneous nature of tumor relapse in groups 3 and 4, there are less specified trials towards those molecular groupings, and radiation seems to be the main scope of treatment. Medulloblastomas being primarily a pediatric tumor require treatment options that minimize radiation to increase the quality of living in children and to prevent long-term symptoms of over radiation. Exploring symptomatic treatment with donepezil in children with combination therapies may be a potential route for future trials; immunotherapies seem to hold potential in treating patients reacting adversely to radiation therapy.

## Introduction and background

Medulloblastomas are classified as WHO grade IV malignant tumors occurring primarily in the cerebellum in pediatric patients; they vary in prognosis depending on the subtype of the tumor. Usually occurring in children under 16, a large majority of patients are under 10 [1].

Pediatric populations diagnosed with medulloblastomas comprise 15%-20% of all brain tumors [2]. Unfortunately, the quality of life for many of these children severely deteriorates despite high survival rates with aggressive chemotherapy, radiotherapy, and neurosurgery [3]. Due to the prevalence of medulloblastomas situated in the posterior fossa, they often rapidly metastasize into the cerebrospinal fluid and affect different parts of the brain, aside from the infratentorial region [4]. Surgical intervention can delay the metastasis of medulloblastomas; however, one or more neurological impairments are a common side effect in 25% of the population. Unfortunately, a familiar condition is posterior fossa syndrome which interferes with communicative activities and manifests into ataxia and hypotonia [5]. Thus, it has become imperative to assess combination therapies that may be used alongside traditional treatment protocols to delay treatment in younger children until they are three and thereby reduce neurological impairment.

The current treatment strategy for medulloblastomas, in children under three, consists primarily of surgery and chemotherapy and yields beneficial results in effectively reducing tumors. The best outcome has been observed with wingless (WNT)-activated medulloblastomas in the cohort of children under 16, but that statistic does not apply to children under five. However, for some subtypes of medulloblastoma, there remain high rates of diffuse metastasis [6]. Treatment in these aggressive situations results in a reduction in quality of survival for patients in terms of decreased intelligence quotient, while neuroendocrine side effects (i.e. growth hormone deficiency and hypothyroidism) generate a necessity for chronic symptomatic treatment [7]. Additionally as previously mentioned, ataxia and hypertonia can be side effects that will generate further medical complications requiring physical therapy, reduced muscle function, and difficulty in performing involuntary actions like swallowing. In this review, we initially focus on the subtypes of medulloblastomas and then review current treatment options, including immunotherapy, stem cell therapy, and pharmacological compounds.

## Review

Types of medulloblastoma

Medulloblastoma tumors are categorized into four subgroups by the Cancer Genome Atlas (WNT, sonic hedgehog (SHH), group 3, and group 4); in spite of current treatments, 30% of patients have a relapse which portends a poor outcome [8]. Any current treatment regimen is not equally effective against the different subgroups of medulloblastoma, and patients that survive past five years may face a recurrence of the disease [9]. Novel treatments are required for individualized subtypes of medulloblastomas since current protocols generalize patients with a similar treatment plan and leave patients with degenerative conditions as a result of long-term treatment toxicity [10].

Group 4

Group 4 medulloblastomas are the most common type and it is prevalent in males three times more frequently than in females [11]. Group 4, SHH-group, and group 3 tumors tend to originate from the intermediary area under the vermis of the cerebellum. Despite its frequency in patients, it is difficult to treat because the tumor metastasizes prior to diagnosis in 30%-40% of patients, which contributes to a low five-year survival rate of 60% [12,13]. Patients with group 3 tumors constitute a high-risk group compared to other sub-types as subtotal resection of the tumor during surgery increases the risk of disease progression, complicating the benefit of gross total or near-total resection [14]. Group 4 tumors have a high rate of recurrence as 30%-40% of patients are at high risk and the five-year survival for children is 60% while for adults there is a high degree of variability with a five-year survival between 45%-75% [15]. The 10-year survival rate is 36% for high-risk group four patients while for low-risk patients it is 72% and this is characterized by their chromosome 11 loss [16].

SHH-Group

SHH group medulloblastomas occur in both infants and adults and are the second most common subgroup. Treatment is a challenge because radiotherapy is highly debilitative in infants under 36 months and adults have a recurrence rate of 50%-60%, regardless of treatment intervention [17, 18]. Adults make for difficult patients to treat as their medulloblastoma genomic profiles are very different in adult medulloblastomas and there is a connection between the amplification of CDK6 and rapidly terminal outcomes [19]. Due to the rarity of adult cases, pediatric regimens tend to be used as treatment protocol and prognosis is not ideal. SHH group tumors are conventionally found in the posterior fossa of the brain in the cerebral hemispheres [20]. This is due to SHH signaling being part of the morphogenesis and maintenance of neurons that form both hemispheres of the brain [21]. SHH-group medulloblastomas tend to happen in infants, and the 10-year survival rate for infants is 77%, children have around 51% success, while for adults it is 35% [22].

Group 3

Group 3 tumors are the most aggressive and metastatic of the sub-types due to the amplification of the MYC gene, which causes tumorigenesis [23-25]. Unfortunately, due to the prognosis of this disease, outside of the conventional treatment of surgery and chemotherapy, there have yet to be targeted treatments developed for group 3 due to the heterogeneity in the nature of tumor recurrence [26]. Current treatments also include craniospinal irradiation for high-risk patients like those with group 3 medulloblastomas, but due to the slowed progression to metastasis, they can be ineffective [27]. 35%-45% of initial group-3 medulloblastoma survivors experience fatal relapse [28]. The 10-year survival rate for this medulloblastoma in infants is 39% and in children, it is 50% [29].

WNT-Group

WNT-activated medulloblastomas have the best prognosis of all the sub-groups; however, they are the least common type of medulloblastoma [30]. WNT signaling, especially when canonical, is associated with many types of cancers [31]. Due to its prevalent nature, the signaling pathway of WNT-activated tumors and metastatic cancers has been thoroughly researched to discover its role in immune evasion. Aberrations in the WNT pathway (i.e., hyperactivation of WNT resulting in medulloblastoma tumors) result in an ideal tumor microenvironment as WNT ligands released by tumor cells bypass the host immune response [32]. Surgery and radiation tend to improve the prognosis for children towards a 10-year survival ≥ 95% when compared to the other subtypes of medulloblastomas [33].

Current therapies available and potential of clinical trials

Standard care of treatment is generally successful to some extent in patients with more common subtypes, and it is primarily a combination of surgery, radiotherapy, and chemotherapy. There are many clinical trials assessing the efficiency of combinations of currently approved treatments, but many patients are more interested in knowing about their clinical trial options, especially at the time of relapse.

For examining the current clinical trials for medulloblastomas, the US government clinical trials website (ClinicalTrials.gov) database was utilized and filtered using the following: medulloblastoma therapies, active, recruiting, enrolling by invitation, and completed studies. The inclusion criteria were comprehensive of both systemic therapies; radiation or surgery-based studies were also considered. Terminated, withdrawn, and unknown status studies were excluded. Of the 82 studies available for medulloblastomas, 32 matched the listed criteria. Treatments with preliminary or interim positive results were grouped based on the type of therapy and mechanisms of action; the preliminary results and the potential of the therapy for medulloblastomas were then summarized and discussed (Table 1).

**Table 1:** Immunotherapy trials for medulloblastomas CAR T cell therapy: chimeric antigen receptor T cell therapy; xALT therapy: autologous lymphocyte transfer; IDO: indoleamine 2,3 dioxygenase; CMV: cytomegalovirus.

Type of Immunotherapy / Vaccines	Mechanism and Current Status	ClinicalTrials.gov Identifier	Citations
CAR T Cell Therapy	FDA approved for aggressive lymphomas, it is being investigated as additive therapy for patients with recurrent medulloblastomas. This therapy uses the patient’s T cells and engineers them to produce chimeric antigen receptors on its surface to identify cancerous cells more efficiently. Therapy in participants of this trial begins after surgical resection, and the current expectation of this new trial is that the B7H3-specific CAR T cells will be able to directly interact with tumor cells due to the procedure using a catheter and may stimulate a positive response.	NCT04185038 NCT03500991 NCT03638167	[34-37]
Adoptive CAR T Therapy for Medulloblastoma Post-Chemotherapy and Stem Cell Transplantation	Using dendritic cell xALT therapy in both recurrent medulloblastoma patients and primitive neuroectodermal tumor patients. Patients enrolled in this study have previously received myeloablative chemotherapy and hematopoietic stem cell transplantation.	NCT01326104	[38]
Indoximod-based immunotherapy with pembrolizumab	Indoximod is an IDO-blocking drug that increases tryptophan levels in order to upregulate T cell function, increasing the immune response to the cancerous cells. Indoximod has shown positive results in Phase I trials and there is a Phase II trial in the status of recruiting.	NCT04049669 NCT02502708	[39,40]
VBI-1901	This enveloped virus-like particle (eVLP) vaccine is designed to target tumors stemming from a cytomegalovirus infection which is the case for the origin of many medulloblastomas and glioblastomas tumors. The VBI-1901 stimulates a patient’s immune system to produce antibodies targeting the tumor cells. Still, in the early stages, it has been granted FDA Fast Track Designation and may soon be heading to clinical trials or in the experimental treatment phase for medulloblastomas.	NCT03382977	[41-43]
CMV-related Vaccine	CMV RNA-pulsed dendritic cell vaccine was used to treat an array of WHO-grade IV tumors. The initial Phase I study was successful and Phase II trials will happen in the future.	NCT03615404	-

Many of the oncolytic virus and vaccine immunotherapies with radiotherapy are still in the trial stage, but it seems that of the three categories, immunotherapies seem to be the future of medulloblastoma treatment [44]. There are many types of viruses that may initiate gliomas, and there is evidence suggesting that measles, myxoma virus, picornavirus, and cytomegalovirus can be involved in the case of medulloblastomas [45-48].

Natural killer cell therapy represents another type of immunotherapy that has been speculated to hold a strong potential for therapy due to strong in vitro results [49]. But the only trial available identified as NCT02271711 does not demonstrate or notify of any results related to the trial [50]. Another therapy, G207 HSV viral therapy or herpes viral therapy with radiation, identified as NCT02457845, demonstrated an increased count for lymphocytes targeting tumor cells. The therapy alone was not effective for aggressively chronic conditions, but combinations can be explored [51] (Table 2).

**Table 2:** Stem cell therapy treatment options for medulloblastomas

Type of Stem Cell Therapy	Current Status	ClinicalTrials.gov Identifier	Citation
Peripheral Stem Cell Transplantation	A few trials are on Phase II or Phase III status indicating that patients do not react adversely to these treatments and many trials reported no toxic deaths. There were a few pediatric medulloblastoma patient survivors, but their sub-type was not diagnosed at the start of the trials. The results were consistent with average life expectancy for large cohort sizes, and it seems that adaptive radiotherapy was more efficient in helping with progress than stem cell transplantations.	NCT00336024 NCT00003211 NCT00003141 NCT00002594 NCT00005952 NCT00003846 NCT00025558	[52-54]

Autologous stem cell rescue (ASCR) therapy, different from induced pluripotent stem cell therapy, uses the patients’ blood stem cells to regrow bone marrow tissue. Usually, this method is used to combat the harsh results of chemotherapy as radiation tends to cause degradation of bone marrow. The efficiency of stem cells at this point in research tends not to provide significant results of progress and is often used in conjunction with other therapies [55] (Table 3).

**Table 3:** Pharmacological treatment for medulloblastomas SHH: sonic hedgehog; ERBB2: erb-b2 receptor tyrosine kinase 2; CNS: erb-b2 receptor tyrosine kinase 2.

Type of Pharmacological Compound (anti-angiogenic therapy, chemotherapy, targeted therapy, etc.)	Compound’s Mechanism & Comments	ClinicalTrials.gov Identifiers	Citation
Vismodegib or GDC-0449	It is an SHH-pathway inhibitor used as targeted therapy for SHH-subtype medulloblastomas. It is used commonly as combination therapy with surgery and chemotherapy for adult medulloblastomas as SHH tends to be the common type in older populations.	NCT00939484 NCT01239316 NCT00822458	[56]
CX-4945	This molecule inhibits CK2 (Casein Kinase 2) which prevents the further proliferation of the cancerous cell. Thus, CK2 inhibitors like CX-4945 are used in many anti-cancer treatments. However, CK2 inhibitors only impact SHH subtype medulloblastomas.	NCT03904862	[57-59]
Bevacizumab and Irinotecan	​​Bevacizumab is a monoclonal antibody drug that is used to treat an array of cancers including glioblastomas and there have been reports that found it to be an effective drug with radiation therapy. Irinotecan is a chemotherapy drug used commonly in colorectal cancer treatments. It is known as a topoisomerase inhibitor that causes cell death by binding to DNA in primarily cancerous cells, but there can be unpredictable side effects in other patients. The combination expects to halt the growth of tumor cells and/or stop the division of further cancer cells. Bevacizumab could prevent growth by preventing a nutrient-rich environment near the tumor and subsequently, irinotecan would prevent cell division in the region.	NCT00381797	[60-63]
Lapatinib ditosylate	Drug targets and block the ERBB2 receptor which is responsible for signaling tumor cell division and growth. The drug did not perform as successfully in medulloblastoma patients compared to other CNS cancers, but it is still successful in breast cancer patients.	NCT00095940	[64]
Temozolomide Combinations (trials with combinations that had discontinued compounds and preliminary unsatisfactory results with not included).	No Combination: Temozolomide This antineoplastic is used to treat different brain tumors and is a very effective drug with a combination of radiation therapy. There has been a positive case report from an adult patient with recurrent medulloblastoma who demonstrated stabilization of tumor growth and reduced symptoms for 8 months with temozolomide. There were initial phase II trials in Italy that saw beneficial progress with temozolomide in children with recurrent medulloblastoma.	NCT00005955	[65-66]
	Combination: ABT-888 (Veliparib) and Temozolomide Mechanism: ABT-888, a poly ADP-ribose inhibitor (expressed excessively in pediatric medulloblastomas), is used to treat other types of cancers, but with temozolomide, it increases the efficiency of targeting tumor cells. Despite initial success in phase I trials, the combination was not successful enough to be considered an efficient protocol for many types of brain tumors non-exclusive to medulloblastomas.	NCT00946335 NCT00994071	[67-68]
Vorinostat Combinations	Combination: Vorinostat and Temozolomide Mechanism: Vorinostat, a histone deacetylase inhibitor, is FDA-approved to use in T-cell lymphomas. The trial anticipated that the combination with Temozolomide may increase the sensitivity of identifying and attacking primary tumor cells in the CNS.	NCT01076530	[69-70]
	Combination: Vorinostat With or Without Isotretinoin Mechanism: Since Vorinostat has shown success in many hematologic malignancies, it was predicted that the compound would inhibit blood flow to the cancerous region and reduce tumor cell division.	NCT00217412	[71]
Irinotecan Combinations	Irinotecan Mechanism: As another antineoplastic drug, irinotecan is a topoisomerase I inhibitor, it blocks the enzyme nuclear DNA topoisomerase and leads to apoptosis of cancerous cells.	NCT00004078	[72-73]
	Oxaliplatin and Irinotecan Mechanism: Oxaliplatin is a neurotoxic chemotherapeutic and has been shown to cause a large portion of patients to demonstrate chemotherapy-induced peripheral neuropathy. The drug is very potent and aggressive and there are not much clinical data on its efficiency for reducing CNS tumor cell growth.	NCT00101270	[74]
Lenalidomide	Pediatric patients with CNS tumors tolerated this drug and short-term toxicity was not seen. However, this compound is effective when increasing the dosage to the maximum of 116 mg/m2/d. In vitro studies have shown that this compound suppresses glioma cell growth and has the potential to be used in brain tumor treatment. Medulloblastoma-specific trials have not been conducted yet.	NCT00100880	[75-76]
Valproic Acid	In broad CNS condition trials determining toxicity, only partial and minor responses were seen in 2 patients out of 16.	NCT00107458	[77]
Donepezil With Previous Radiation Therapy	Donepezil is primarily used to treat symptoms of dementia in patients with Alzheimer’s by replenishing neurotransmitters. One Phase III trial results demonstrated positive maintenance of memory, motor speed, and dexterity in patients with CNS tumors. A pilot study assessing the effects on pediatric brain tumors has favorable findings as cognitive functions showed an improvement.	NCT00452868	[78-79]
Arsenic Trioxide	Arsenic trioxide acts as a radiosensitizer in solid tumors, and this result has been seen with pediatric SHH-subtype cell lines. This treatment holds potential for becoming protocol as it increases the efficiency of conventional therapy for other types of tumors.	NCT00024258	[80]
Busulfan	Busulfan is an alkylating agent that attaches to DNA strands of the cancerous cell and prevents further division. There have been positive results in some trials. One of which saw no toxic death but saw hepatic veno-occlusive disease which was manageable.	NCT00006246	[81-82]

**Figure 1:**
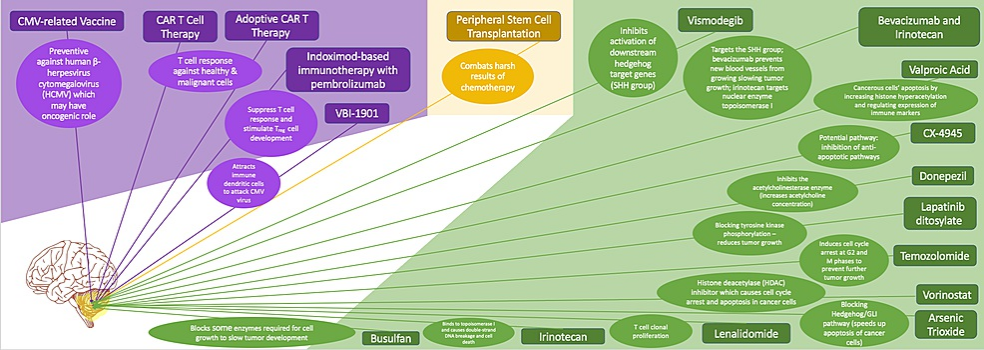
Comprehensive diagram of molecular mechanisms of potential therapies CMV: cytomegalovirus; CAR T cell therapy: chimeric antigen receptor T cell therapy. The image was created by the authors.

Many of the trials solely used one pharmacological compound paired with surgical resection and radiation therapy. The type of radiation therapy available at the location and time of the trials may have affected the results, but rather than that, combination therapies seemed to be ideal for some trials. The mechanism of many of the drugs targets SHH-subtype medulloblastomas and cannot be used in broad-spectrum therapies. For the increasing quality of life, donepezil seems to be a drug that may be useful for early intervention to delay symptoms in children under the age of five, however, the success of donepezil may be due to the combination with radiation. Donepezil is a cholinesterase inhibitor that increases the concentration of neurotransmitter acetylcholine in the brain, further inducing synaptic plasticity in the brain. Often relapses in WNT-activated medulloblastomas are a result of continuous aggressive treatment regimens which cause an accumulation of cyclophosphamide doses, but this is being improved with a stronger emphasis on abatement of both chemotherapy and radiation [83]. Other pharmacological interventions need to be used on a case-by-case basis to identify suitable or unsuitable combinations (Figure 1).

Discussion

From analyzing the results and current statuses of clinical trials associated with functional medulloblastoma treatment, it is plausible to state that SHH-subtypes are the most targeted; recurrent medulloblastoma patients are also a target. Children under three tend to be neglected for treatment options as combination therapies with radiation provide ideal outcomes. Immunotherapies with combination treatments may be suitable for protocol treatments for patients with specific profiles, and pharmacological compounds or stem cell therapy may be potential treatment avenues with promising results when done with conventional surgical resection and radiation therapy. These treatment options may also allow for children under three to avoid radiation therapy until they are of age.

Many of the treatments and clinical trials aim to target a plethora of CNS tumors rather than specifically targeting medulloblastomas. This may be due to the rarity of the condition or the lack of participation from the small subset of patients in experimental clinical trials, but this reduces the overall specificity of treatment for different types of medulloblastomas. Many completed clinical trials also did not report their study results in the form of a publication or data in the clinicaltrials.gov website which may indicate unfavorable data results. 

When comparing the studies, it is essential to realize that combination therapies hold the most promise in these experimental treatment plans. There are some forward therapies which are used in older populations like vismodegib and others which simply target the SHH-group medulloblastomas and that can be seen with CX-4945 and arsenic trioxide in addition to vismodegib for pharmaceutical therapies. Immunotherapy holds promise as there is a genetic component of medulloblastomas; training the innate system to destroy the cancer cells, in addition to surgical resection and adaptive radiotherapy, seems to hold the most potential for widespread intervention protocol development as they attempt to minimize the complications that many pharmaceutical reagents can bring. Donepezil may also be helpful in pediatric patients who require radiation minimization for the quality of future development, but radiotherapy becomes essential with any of the therapy options offered in trials.

## Conclusions

It is important to investigate individualized treatment plans in reference to the sub-type of medulloblastoma targeted. Future studies should also be comprehensive of international clinical studies as many data-recording platforms are not translated or exclusively demonstrate national studies. Doing so would allow for more clinical trial options for patients and their families. Children who are medulloblastoma survivors have a severe need for symptomatic treatment to preserve their cognitive and neuroendocrine functions. Since the pertinent issue is increasing length of survival, there should also be trials investigating the quality of survival through methods of combating impaired cognition and reducing radiotherapy. Since medulloblastoma treatment, especially when recurring, is mainly focused on increasing the quality of living while extending life expectancy, there are caveats in available treatments; experimental therapy consent becomes a sensitive issue for families when treating children. Prospective successful treatments may be a combination of chemotherapy, radiation, and immunotherapy post-surgical resection and the combination of chemotherapy and immunotherapy could be a sustained solution for children without radiation. This would allow many medulloblastoma survivors to have a more cognizant and independent experience of living as their developmental functions would not be toxically affected.

## References

[1] (2022). Medulloblastoma - childhood statistics. https://www.cancer.net/cancer-types/medulloblastoma-childhood/statistics.

[2] Rossi A, Caracciolo V, Russo G, Reiss K, Giordano A (2008). Medulloblastoma: from molecular pathology to therapy. Clin Cancer Res.

[3] de Medeiros CB, Moxon-Emre I, Scantlebury N (2020). Medulloblastoma has a global impact on health related quality of life: findings from an international cohort. Cancer Med.

[4] Zapotocky M, Mata-Mbemba D, Sumerauer D (2018). Differential patterns of metastatic dissemination across medulloblastoma subgroups. J Neurosurg Pediatr.

[5] Schepke E, Tisell M, Kennedy C (2020). Effects of the growth pattern of medulloblastoma on short-term neurological impairments after surgery: results from the prospective multicenter HIT-SIOP PNET 4 study. J Neurosurg Pediatr.

[6] Hill RM, Richardson S, Schwalbe EC (2020). Time, pattern, and outcome of medulloblastoma relapse and their association with tumour biology at diagnosis and therapy: a multicentre cohort study. Lancet Child Adolesc Health.

[7] Kline CN, Packer RJ, Hwang EI (2017). Case-based review: pediatric medulloblastoma. Neurooncol Pract.

[8] Sharma T, Schwalbe EC, Williamson D (2019). Second-generation molecular subgrouping of medulloblastoma: an international meta-analysis of Group 3 and Group 4 subtypes. Acta Neuropathol.

[9] Warmuth-Metz M, Blashofer S, von Bueren AO (2011). Recurrence in childhood medulloblastoma. J Neurooncol.

[10] De B, Beal K, De Braganca KC (2018). Long-term outcomes of adult medulloblastoma patients treated with radiotherapy. J Neurooncol.

[11] Northcott PA, Korshunov A, Pfister SM, Taylor MD (2012). The clinical implications of medulloblastoma subgroups. Nat Rev Neurol.

[12] Ramaswamy V, Remke M, Bouffet E (2016). Risk stratification of childhood medulloblastoma in the molecular era: the current consensus. Acta Neuropathol.

[13] Thompson EM, Hielscher T, Bouffet E (2016). Prognostic value of medulloblastoma extent of resection after accounting for molecular subgroup: a retrospective integrated clinical and molecular analysis. Lancet Oncol.

[14] Menyhárt O, Giangaspero F, Győrffy B (2019). Molecular markers and potential therapeutic targets in non-WNT/non-SHH (group 3 and group 4) medulloblastomas. J Hematol Oncol.

[15] Schwalbe EC, Lindsey JC, Nakjang S (2017). Novel molecular subgroups for clinical classification and outcome prediction in childhood medulloblastoma: a cohort study. Lancet Oncol.

[16] Taylor MD, Northcott PA, Korshunov A (2012). Molecular subgroups of medulloblastoma: the current consensus. Acta Neuropathol.

[17] Prados MD, Warnick RE, Wara WM (1995). Medulloblastoma in adults. Int J Radiat Oncol Biol Phys.

[18] Korshunov A, Remke M, Werft W (2010). Adult and pediatric medulloblastomas are genetically distinct and require different algorithms for molecular risk stratification. J Clin Oncol.

[19] Sengupta S, Pomeranz Krummel D, Pomeroy S (2017). The evolution of medulloblastoma therapy to personalized medicine. F1000Res.

[20] Li X, Li Y, Li S, Li H, Yang C, Lin J (2021). The role of Shh signalling pathway in central nervous system development and related diseases. Cell Biochem Funct.

[21] AlRayahi J, Zapotocky M, Ramaswamy V (2018). Pediatric brain tumor genetics: what radiologists need to know. Radiographics.

[22] Cho YJ, Tsherniak A, Tamayo P (2011). Integrative genomic analysis of medulloblastoma identifies a molecular subgroup that drives poor clinical outcome. J Clin Oncol.

[23] Kool M, Korshunov A, Remke M (2012). Molecular subgroups of medulloblastoma: an international meta-analysis of transcriptome, genetic aberrations, and clinical data of WNT, SHH, Group 3, and Group 4 medulloblastomas. Acta Neuropathol.

[24] Northcott PA, Robinson GW, Kratz CP (2019). Medulloblastoma. Nat Rev Dis Primers.

[25] Gabay M, Li Y, Felsher DW (2014). MYC activation is a hallmark of cancer initiation and maintenance. Cold Spring Harb Perspect Med.

[26] Schwinn S, Mokhtari Z, Thusek S (2021). Cytotoxic effects and tolerability of gemcitabine and axitinib in a xenograft model for c-myc amplified medulloblastoma. Sci Rep.

[27] Martirosian V, Neman J (2018). Medulloblastoma: challenges and advances in treatment and research. Cancer Rep.

[28] Stock A, Mynarek M, Pietsch T (2019). Imaging characteristics of wingless pathway subgroup medulloblastomas: results from the German HIT/SIOP-trial cohort. AJNR Am J Neuroradiol.

[29] Endo M, Nishita M, Fujii M, Minami Y (2015). Insight into the role of Wnt5a-induced signaling in normal and cancer cells. Int Rev Cell Mol Biol.

[30] Jaiswal R, Johnson MS, Pokharel D, Krishnan SR, Bebawy M (2017). Microparticles shed from multidrug resistant breast cancer cells provide a parallel survival pathway through immune evasion. BMC Cancer.

[31] Nalita N, Ratanalert S, Kanjanapradit K, Chotsampancharoen T, Tunthanathip T (2018). Survival and prognostic factors in pediatric patients with medulloblastoma in southern Thailand. J Pediatr Neurosci.

[32] Gajjar A, Chintagumpala M, Ashley D (2006). Risk-adapted craniospinal radiotherapy followed by high-dose chemotherapy and stem-cell rescue in children with newly diagnosed medulloblastoma (St Jude Medulloblastoma-96): long-term results from a prospective, multicentre trial. Lancet Oncol.

[33] (2022). KYMRIAH (tisagenlecleucel). https://www.fda.gov/vaccines-blood-biologics/cellular-gene-therapy-products/kymriah-tisagenlecleucel.

[34] Donovan LK, Delaidelli A, Joseph SK (2020). Locoregional delivery of CAR T cells to the cerebrospinal fluid for treatment of metastatic medulloblastoma and ependymoma. Nat Med.

[35] Benmebarek MR, Karches CH, Cadilha BL, Lesch S, Endres S, Kobold S (2019). Killing mechanisms of chimeric antigen receptor (CAR) T cells. Int J Mol Sci.

[36] (2022). Study of B7-H3-specific CAR T cell locoregional immunotherapy for diffuse intrinsic pontine glioma/diffuse midline glioma and recurrent or refractory pediatric central nervous system tumors. https://clinicaltrials.gov/ct2/show/NCT04185038.

[37] (2022). Vaccine immunotherapy for recurrent medulloblastoma and primitive neuroectodermal tumor. https://clinicaltrials.gov/ct2/show/NCT01326104.

[38] Fox E, Oliver T, Rowe M (2018). Indoximod: an immunometabolic adjuvant that empowers T cell activity in cancer. Front Oncol.

[39] (2022). Pediatric trial of indoximod with chemotherapy and radiation for relapsed brain tumors or newly diagnosed dipg. https://clinicaltrials.gov/ct2/show/study/NCT04049669.

[40] (2022). VBI vaccines announces positive interim phase 2a data from VBI-1901 in recurrent gbm. https://www.vbivaccines.com/posters/sno-2020-phase-2a-data/.

[41] Söderberg-Nauclér C, Johnsen JI (2015). Cytomegalovirus in human brain tumors: role in pathogenesis and potential treatment options. World J Exp Med.

[42] (2022). VBI vaccines granted FDA fast track designation for VBI-1901 for the treatment of recurrent gbm. https://www.vbivaccines.com/press-releases/vbi-1901-gbm-fda-fast-track-designation/.

[43] Kabir TF, Kunos CA, Villano JL, Chauhan A (2020). Immunotherapy for medulloblastoma: current perspectives. Immunotargets Ther.

[44] Studebaker AW, Kreofsky CR, Pierson CR, Russell SJ, Galanis E, Raffel C (2010). Treatment of medulloblastoma with a modified measles virus. Neuro Oncol.

[45] Lun XQ, Zhou H, Alain T (2007). Targeting human medulloblastoma: oncolytic virotherapy with myxoma virus is enhanced by rapamycin. Cancer Res.

[46] Yu L, Baxter PA, Zhao X (2011). A single intravenous injection of oncolytic picornavirus SVV-001 eliminates medulloblastomas in primary tumor-based orthotopic xenograft mouse models. Neuro Oncol.

[47] Baryawno N, Rahbar A, Wolmer-Solberg N (2011). Detection of human cytomegalovirus in medulloblastomas reveals a potential therapeutic target. J Clin Invest.

[48] Powell AB, Yadavilli S, Saunders D (2019). Medulloblastoma rendered susceptible to NK-cell attack by TGFβ neutralization. J Transl Med.

[49] (2022). Expanded natural killer cell infusion in treating younger patients with recurrent/refractory brain tumors. https://clinicaltrials.gov/ct2/show/NCT02271711.

[50] (2022). Abstract CT216: Phase I study of intraventricular infusions of autologous ex vivo expanded NK cells in children with recurrent/refractory malignant posterior fossa tumors of the central nervous system. https://cancerres.aacrjournals.org/content/79/13_Supplement/CT216.article-info.

[51] Friedman GK, Johnston JM, Bag AK (2021). Oncolytic HSV-1 G207 immunovirotherapy for pediatric high-grade gliomas. N Engl J Med.

[52] Kadota RP, Mahoney DH, Doyle J (2008). Dose intensive melphalan and cyclophosphamide with autologous hematopoietic stem cells for recurrent medulloblastoma or germinoma. Pediatr Blood Cancer.

[53] Laughton SJ, Merchant TE, Sklar CA (2008). Endocrine outcomes for children with embryonal brain tumors after risk-adapted craniospinal and conformal primary-site irradiation and high-dose chemotherapy with stem-cell rescue on the SJMB-96 trial. J Clin Oncol.

[54] Sadanandan N, Shear A, Brooks B (2021). Treating metastatic brain cancers with stem cells. Front Mol Neurosci.

[55] Lou E, Schomaker M, Wilson JD, Ahrens M, Dolan M, Nelson AC (2016). Complete and sustained response of adult medulloblastoma to first-line sonic hedgehog inhibition with vismodegib. Cancer Biol Ther.

[56] Climans SA, Macdonald DR, Sutherland DE, Mason WP (2020). Prolonged response to vismodegib in a patient with systemic medulloblastoma metastases. BMJ Case Rep.

[57] Nitta RT, Bolin S, Luo E (2019). Casein kinase 2 inhibition sensitizes medulloblastoma to temozolomide. Oncogene.

[58] Chon HJ, Bae KJ, Lee Y, Kim J (2015). The casein kinase 2 inhibitor, CX-4945, as an anti-cancer drug in treatment of human hematological malignancies. Front Pharmacol.

[59] Purzner T, Purzner J, Buckstaff T (2018). Developmental phosphoproteomics identifies the kinase CK2 as a driver of Hedgehog signaling and a therapeutic target in medulloblastoma. Sci Signal.

[60] Zhao M, Wang X, Fu X, Zhang Z (2018). Bevacizumab and stereotactic radiosurgery achieved complete response for pediatric recurrent medulloblastoma. J Cancer Res Ther.

[61] Hsiang YH, Lihou MG, Liu LF (1989). Arrest of replication forks by drug-stabilized topoisomerase I-DNA cleavable complexes as a mechanism of cell killing by camptothecin. Cancer Res.

[62] Rothenberg ML, Meropol NJ, Poplin EA, Van Cutsem E, Wadler S (2001). Mortality associated with irinotecan plus bolus fluorouracil/leucovorin: summary findings of an independent panel. J Clin Oncol.

[63] Han K, Peyret T, Quartino A (2016). Bevacizumab dosing strategy in paediatric cancer patients based on population pharmacokinetic analysis with external validation. Br J Clin Pharmacol.

[64] Cruickshanks N, Hamed HA, Bareford MD, Poklepovic A, Fisher PB, Grant S, Dent P (2012). Lapatinib and obatoclax kill tumor cells through blockade of ERBB1/3/4 and through inhibition of BCL-XL and MCL-1. Mol Pharmacol.

[65] Poelen J, Bernsen HJ, Prick MJ (2007). Metastatic medulloblastoma in an adult; treatment with temozolomide. Acta Neurol Belg.

[66] Cefalo G, Massimino M, Ruggiero A (2014). Temozolomide is an active agent in children with recurrent medulloblastoma/primitive neuroectodermal tumor: an Italian multi-institutional phase II trial. Neuro Oncol.

[67] Su JM, Thompson P, Adesina A (2014). A phase I trial of veliparib (ABT-888) and temozolomide in children with recurrent CNS tumors: a pediatric brain tumor consortium report. Neuro Oncol.

[68] Donawho CK, Luo Y, Luo Y (2007). ABT-888, an orally active poly(ADP-ribose) polymerase inhibitor that potentiates DNA-damaging agents in preclinical tumor models. Clin Cancer Res.

[69] (2022). Vorinostat and temozolomide in treating young patients with relapsed or refractory primary brain tumors or spinal cord tumors. https://clinicaltrials.gov/ct2/show/NCT01076530.

[70] Siegel D, Hussein M, Belani C (2009). Vorinostat in solid and hematologic malignancies. J Hematol Oncol.

[71] (2022). Vorinostat with or without Isotretinoin in treating young patients with recurrent or refractory solid tumors, lymphoma, or leukemia. https://www.clinicaltrials.gov/ct2/show/study/NCT00217412.

[72] Fujita K, Kubota Y, Ishida H, Sasaki Y (2015). Irinotecan, a key chemotherapeutic drug for metastatic colorectal cancer. World J Gastroenterol.

[73] Pommier Y (2006). Topoisomerase I inhibitors: camptothecins and beyond. Nat Rev Cancer.

[74] Selvy M, Pereira B, Kerckhove N (2020). Long-term prevalence of sensory chemotherapy-induced peripheral neuropathy for 5 years after adjuvant FOLFOX chemotherapy to treat colorectal cancer: a multicenter cross-sectional study. J Clin Med.

[75] Warren KE, Goldman S, Pollack IF (2011). Phase I trial of lenalidomide in pediatric patients with recurrent, refractory, or progressive primary CNS tumors: Pediatric Brain Tumor Consortium study PBTC-018. J Clin Oncol.

[76] Hanashima Y, Sano E, Sumi K (2020). Antitumor effect of lenalidomide in malignant glioma cell lines. Oncol Rep.

[77] Su JM, Li XN, Thompson P (2011). Phase 1 study of valproic acid in pediatric patients with refractory solid or CNS tumors: a children's oncology group report. Clin Cancer Res.

[78] Rapp SR, Case LD, Peiffer A (2015). Donepezil for irradiated brain tumor survivors: a phase III randomized placebo-controlled clinical trial. J Clin Oncol.

[79] Castellino SM, Tooze JA, Flowers L, Hill DF, McMullen KP, Shaw EG, Parsons SK (2012). Toxicity and efficacy of the acetylcholinesterase (AChe) inhibitor donepezil in childhood brain tumor survivors: a pilot study. Pediatr Blood Cancer.

[80] Dos Santos Klinger PH, Delsin LE, Cruzeiro GA (2020). Arsenic trioxide exerts cytotoxic and radiosensitizing effects in pediatric medulloblastoma cell lines of shh subgroup. Sci Rep.

[81] Patel R, Tadi P (2021). Busulfan. https://www.ncbi.nlm.nih.gov/books/NBK555986/.

[82] Bergthold G, El Kababri M, Varlet P (2014). High-dose busulfan-thiotepa with autologous stem cell transplantation followed by posterior fossa irradiation in young children with classical or incompletely resected medulloblastoma. Pediatr Blood Cancer.

[83] Nobre L, Zapotocky M, Khan S (2020). Pattern of relapse and treatment response in WNT-activated medulloblastoma. Cell Rep Med.

